# Antidiabetic Drug Prescription Pattern in Hospitalized Older Patients with Diabetes

**DOI:** 10.3390/ijerph20032607

**Published:** 2023-01-31

**Authors:** Ilaria Ardoino, Sara Mandelli, Marta Baviera, Raffaella Rossio, Alessandro Nobili, Pier Mannuccio Mannucci, Carlotta Franchi, on behalf of REPOSI Investigator

**Affiliations:** 1 Laboratory of Pharmacoepidemiology and Human Nutrition, Department of Health Policy, Istituto di Ricerche Farmacologiche Mario Negri IRCCS, 20156 Milan, Italy; 2 Laboratory of Cardiovascular Prevention, Department of Health Policy, Istituto di Ricerche Farmacologiche Mario Negri IRCCS, 20156 Milan, Italy; 3 Department of Pathophysiology and Transplantation, Fondazione IRCCS Cà Granda Ospedale Maggiore Policlinico, 20122 Milan, Italy; 4 Angelo Bianchi Bonomi Hemophilia and Thrombosis Center, Fondazione IRCCS Cà Granda Ospedale Maggiore Policlinico, 20122 Milan, Italy; 5 Italian Institute For Planetary Health (IIPH), 20156 Milan, Italy

**Keywords:** older patients, hospital setting, antidiabetic drug, cardiovascular drug

## Abstract

Objective: To describe the prescription pattern of antidiabetic and cardiovascular drugs in a cohort of hospitalized older patients with diabetes. Methods: Patients with diabetes aged 65 years or older hospitalized in internal medicine and/or geriatric wards throughout Italy and enrolled in the REPOSI (REgistro POliterapuie SIMI—Società Italiana di Medicina Interna) registry from 2010 to 2019 and discharged alive were included. Results: Among 1703 patients with diabetes, 1433 (84.2%) were on treatment with at least one antidiabetic drug at hospital admission, mainly prescribed as monotherapy with insulin (28.3%) or metformin (19.2%). The proportion of treated patients decreased at discharge (*N* = 1309, 76.9%), with a significant reduction over time. Among those prescribed, the proportion of those with insulin alone increased over time (*p* = 0.0066), while the proportion of those prescribed sulfonylureas decreased (*p* < 0.0001). Among patients receiving antidiabetic therapy at discharge, 1063 (81.2%) were also prescribed cardiovascular drugs, mainly with an antihypertensive drug alone or in combination (*N* = 777, 73.1%). Conclusion: The management of older patients with diabetes in a hospital setting is often sub-optimal, as shown by the increasing trend in insulin at discharge, even if an overall improvement has been highlighted by the prevalent decrease in sulfonylureas prescription.

## 1. Introduction

Diabetes mellitus (DM) is a chronic metabolic disorder characterized by hyperglycemia resulting from defects in insulin secretion, insulin function or both and requires continuous medical care. More than 95% of people with diabetes suffer from type 2 diabetes (T2DM) [1]. This condition may occur at any age, but most frequently arises in middle older age and is largely the result of an unhealthy lifestyle, such as tobacco smoking, inadequate diet and lack of physical activity leading to excess body weight or obesity [1].

It has been estimated that nearly 537 million adults (20–79 years) worldwide live with diabetes and that approximately 6.7 million have died due to diabetes or its complications in 2021, with large differences across different geographical areas [2]. In the same year, the age-adjusted prevalence of diabetes in Europe was about 7.0%, with the highest rate in Turkey and Spain and the lowest in Ireland, while Italy’s rate was 6.4%, which was slightly lower than the European average [2]. Moreover, the proportion of people with undiagnosed diabetes was surprisingly high; about one out of two adults with diabetes were unaware of their condition, with higher percentages across countries with low access to healthcare services [2,3]. In light of the aging population worldwide, this makes diabetes a public health global challenge, with considerable high costs in terms of disabilities and health expenditure.

All in all, more than a quarter of people aged 65 years or more live with diabetes [2]. In this sector of the population, diabetes is frequently accompanied by multiple comorbidities; at least one comorbidity in 60% of cases and four or more in 40% of older people with diabetes [4]; subsequently, this may lead to severe complications and to hospitalizations [5]. Many studies have shown two- to three-fold higher rates of hospital admissions among older patients with T2DM in relation to the general population and longer lengths of hospital stay [6].

In general, according to the current clinical guidelines for diabetes, older adults presenting in good general condition may be managed similarly to younger adults [7,8]. Firstly, a healthy diet and lifestyle are mandatory for better glycemic control, while the beginning of pharmacological treatment is recommended when lifestyle alone is unable to maintain the target glycemic control [8]. Among pharmacologic agents, metformin, unless contraindicated, has the strongest evidence of long-term safety and efficacy and therefore was recommended as the first line of treatment.

In recent years, novel classes of antidiabetic drugs have been introduced in clinical practice due to their good efficacy and safety profile. In particular, glucagon-like peptide-1 receptor agonists (GLP-1-RA) and sodium-glucose co-transporter-2 inhibitors (SGLT-2i) have shown additional benefits in terms of weight loss and blood pressure reduction. This class of agents has also been found to be beneficial for patients with heart failure and to slow the progression of chronic kidney disease, making them particularly attractive for patients with established cardiovascular disease and/or with impaired renal function.

The 2022 American Diabetes Association (ADA) standards of medical care in diabetes include GLP-1-RA as the initial pharmacological therapy for T2DM patients with atherosclerotic cardiovascular disease or obesity and SGLT2-i (alone or in combination with metformin) in those with chronic kidney disease, cardiovascular disease or heart failure [7]. Intensifications of antidiabetic treatment with oral antidiabetic drugs in combination and/or with insulin should be carefully evaluated and limited to inadequately controlled patients [7].

Pertaining to the hospital setting, in the absence of controlled clinical trials or even observational data regarding how best to manage hospitalized patients with diabetes, the management approach is based primarily upon clinical expertise. Acute hyperglycemia is common in hospitalized patients, both those with and those without recognized diabetes, and basal plus bolus insulin therapy is emerging as the optimal treatment strategy [8,9].

Diabetes is also associated with long-term damage to multiple organs and patients with diabetes had a two- to four-times increased risk of developing cardiovascular diseases (CVD) with respect to the general population due to both microvascular and macrovascular complications. CVD, with a prevalence of about 32.2%, is the most prevalent cause of morbidity and mortality in patients with DM [10,11]. Many lifestyle-related risk factors (e.g., obesity, hypertension, and dyslipidemia) for CVD are particularly common in patients with T2DM and may further contribute to the development of CVD. This issue solicited many national and international guidelines to emphasize the need to reduce cardiovascular (CV) risk in patients with diabetes by means of pharmacological therapy, mainly with antihypertensives, lipid lowering drugs and antiplatelet agents [7,12,13].

With this background, the aims of this work were to describe (1) the pattern of antidiabetic drugs at hospital admission and discharge, (2) their changes over time, and (3) the co-prescription with other cardiovascular drugs for the prevention and/or treatment of CV complications in older patients with diabetes enrolled from 2010 to 2019 in the REPOSI (REgistro POliterapuie SIMI—Società Italiana di Medicina Interna) registry.

## 2. Materials and Methods

### 2.1. Setting

We obtained data from the REPOSI registry. REPOSI is a multicenter, prospective registry promoted by the Italian Society of Internal Medicine (SIMI), the Fondazione IRCCS Cà Granda Ospedale Maggiore Policlinico and the Istituto di Ricerche Farmacologiche Mario Negri IRCCS in Milan that involves about 100 internal medicine and geriatrics hospital wards throughout Italy and collects clinical and therapeutic information from in-patients aged 65 years or older [14,15]. The database includes information about socio-demographic characteristics, activities of daily living according to the Barthel Index [16], co-morbidities according to the Cumulative Illness Rating Scale (CIRS) [17] and drugs prescribed at admission, during hospital stay and at discharge. REPOSI was approved by the Ethics Committee of the IRCCS Fondazione Ca’ Granda Ospedale Maggiore Policlinico and then by the local committees of the participating wards. This study was conducted following Good Clinical Practices and the Declaration of Helsinki. All patients enrolled in the study provided signed informed consent.

Diseases were classified according to the International Classification of Diseases, 9th revision, Clinical Modification (ICD9-CM), and drugs were coded using the Anatomical Therapeutic Chemical Classification (ATC) system.

### 2.2. Study Population

Patients participating in the REPOSI from 2010 to 2019 were included in this study if, at hospital admission, they: (1) had a diagnosis of diabetes (ICD9-CM code: 250.*) or at least a prescription of insulin or of another antidiabetic drug (AD) (ATC code: A10*) and (2) were discharged alive from hospital.

The following classes of cardiovascular drugs were also considered in the analysis: angiotensin-converting enzyme inhibitors—ACE-I; angiotensin receptor blockers—ARBs (ATC: C09); lipid-lowering drugs (ATC: C10) and antiplatelet drugs (ATC: B01AC).

### 2.3. Statistical Analysis

Characteristics of patients with diabetes were described using frequencies and percentages for categorical variables and mean and standard deviation (SD) or median and interquartile range (IQR) for continuous variables, as appropriate.

The Cochrane–Armitage test was used to test the prescribing patterns of antidiabetic and cardiovascular drugs over time. Statistical analyses were conducted using SAS 9.4.

## 3. Results

### 3.1. Study Population

**Figure 1:**
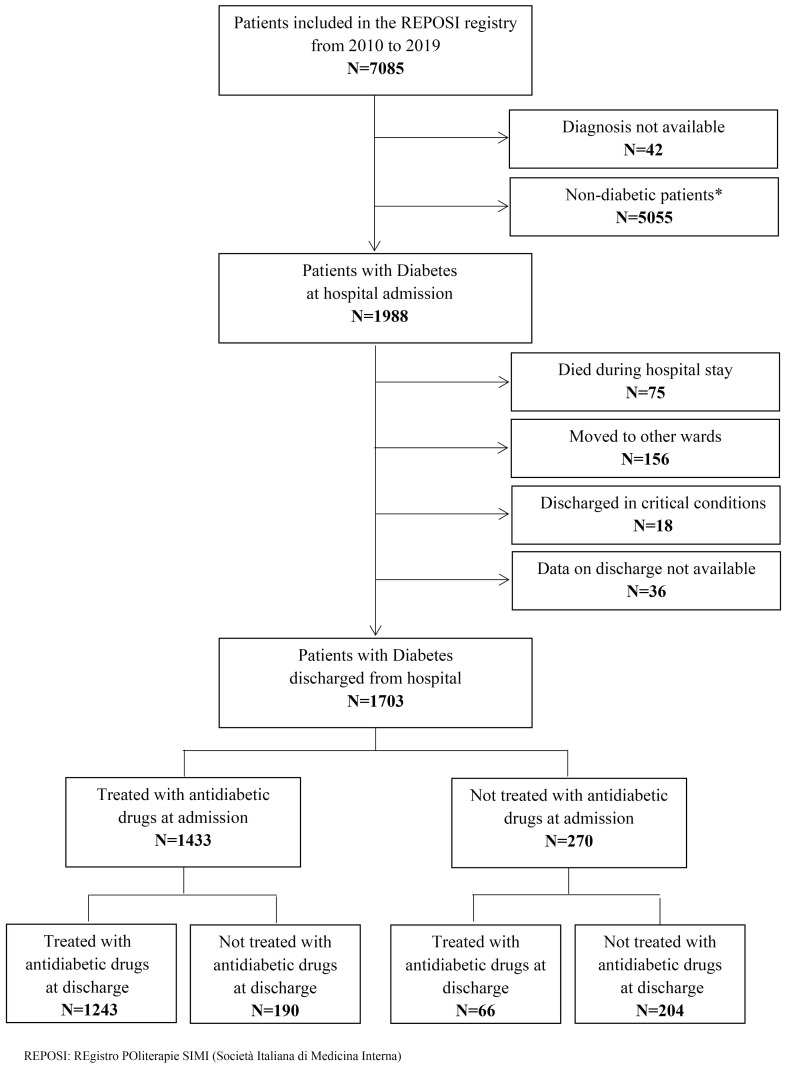
Flow-chart of the patients included in the study from 2010 to 2019. * Patients with Diabetes were identified as those with a diagnosis of diabetes at admission (International Classification of Disease 9-Clinical Modification (ICD-9-CM) code: 250.*) or prescribed with an antidiabetic drug (Anatomical Therapeutic Chemical (ATC) Classification Code = A10).

Figure 1 reports the flow-chart of patients included in the study. From 2010 to 2019, 7085 patients were enrolled in the REPOSI registry. Among them, 1988 patients were identified as those with diabetes if they had a diagnosis of diabetes at hospital admission or were prescribed with at least one antidiabetic drug. In all, 285 patients were not assessable at hospital discharge, thus 1703 patients were included in the present analysis, of them 1689 (99.2%) had a T2DM diagnosis.

Table 1 reports the main sociodemographic characteristics and comorbidities of the 1703 patients. A slight excess in numbers of males was found (53.4% vs. 46.6%) among patients with diabetes and a mean age of 78 (SD 7) years old. Hypertension was the most frequent comorbidity, affecting about 75% of patients (*N* = 1274). In all, 988 (68.0%) patients presented with at least one additional CVD (e.g., ischemic heart disease, heart failure, atrial fibrillation). The causes of hospital admissions are reported in Table S1 (Supplementary Materials).

### 3.2. Antidiabetic Drugs

Among 1703 patients with diabetes, 1433 (84.2%) were on treatment with at least an antidiabetic drug (AD) at hospital admission. Among 1433 patients given a prescription, the most frequently prescribed drugs, alone or in combination, were insulin (*N* = 623, 43.5%), followed by metformin (*N* = 538, 37.5%) and sulfonylureas (*N* = 212, 14.8%). Table 2 reports the prescription pattern of antidiabetic drugs both at hospital admission and discharge.

Most patients were on monotherapy (*N* = 1054/1433, 73.6%). In all, 379 of 1433 patients (26.4%) were prescribed with combinations; 141 (37.2%) of them received insulin in combination with other AD, mainly with metformin (*N* = 64). The proportion of patients prescribed with at least one AD slightly decreased at hospital discharge with respect to hospital admission. Among 1433 patients given a prescription, 190 (13.3%) withdrew from the treatment, while of 270 patients not given a prescription, 66 (24.4%) were newly prescribed, so 1309 of 1703 (76.9%) were prescribed at hospital discharge (Figure 1 and Table 2).

Among 1309 patients given a prescription at hospital discharge, the proportion of those prescribed insulin (alone or in combination) increased from 43.5% to 55.2%, while that of those using metformin and sulfonylureas decreased from 37.5% to 31.7% and from 14.8% to 9.9%, respectively.

Tables S2 and S3 show how the prescription pattern changed over time at hospital admission and discharge. There was a significant reduction in the proportion of patients prescribed AD at hospital discharge. Among patients given a prescription, the proportion of those prescribed insulin alone increased up to 2017 and slightly decreased in the last two years. Moreover, it decreased the proportion of patients prescribed sulfonylureas alone or in combination (with a reduction of 63.3%, *p* < 0.0001). Sulfonylureas were also less prescribed at hospital admission, although with a smaller variation (−44.4%, *p* = 0.0004).

### 3.3. Concomitant Cardiovascular Therapies

**Figure 2:**
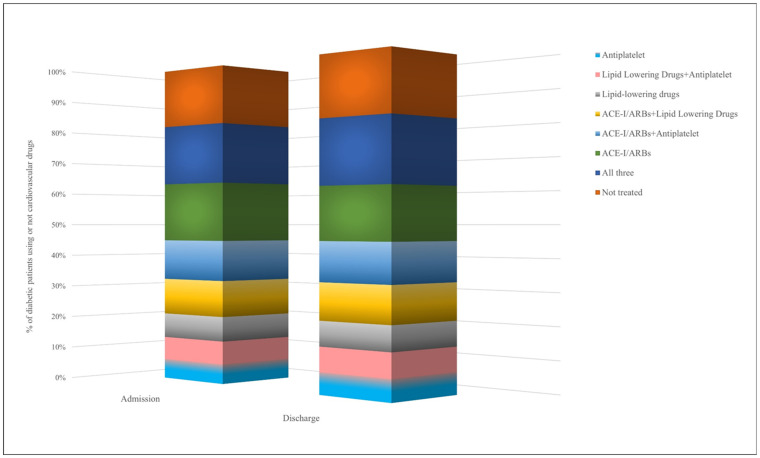
Prevalence of patients with diabetes prescribed or not cardiovascular drugs.

Among patients with diabetes receiving AD at hospital discharge, 1063 (81.2%) were also prescribed the considered cardiovascular drugs, most of them being prescribed an antihypertensive drug (i.e., angiotensin-converting enzyme inhibitors—ACE-I or angiotensin receptor blockers—ARBs) alone or in combination (*N* = 777, 73.1%). In all, 259 patients (24.4%) were prescribed all three of the cardiovascular drugs considered (Figure 2).

No appreciable trend could be observed over time, but prescription of antihypertensive and of antiplatelet slightly decreased in the last two years (from 60.8% in 2010 to 52.4% in 2019 and from 49.7% to 35.9%, respectively), and, on the contrary, statins slightly increased (from 40.6% in 2010 to 50% in 2019) (Tables S4 and S5). Among patients prescribed an antiplatelet drug at hospital discharge, nearly 70% were in secondary prevention due to a previous cardio- or cerebro-vascular event (*N* = 414).

## 4. Discussion

Our study was set up to investigate the management of hospitalized older patients with diabetes. In our cohort, about 85% of patients took at least one medication for diabetes at hospital admission, mainly insulin or metformin. Overall, the prevalence of patients prescribed with AD slightly decreased (in about 9%) at hospital discharge, while the prescription of insulin, alone or in combination, increased. The use of sulfonylureas decreased over time at hospital admission, but much more so at hospital discharge. About 80% of patients with diabetes were also co-prescribed with CV drugs at hospital discharge, mainly with ACE inhibitors (alone or in combination).

T2DM represents the most common metabolic disease in older adults, and it is recognized as being among the most important causes of premature death and disability [18,19].

Older people are often characterized by multiple coexisting comorbidities and are frequently exposed to polypharmacy, which is associated with an increased risk of adverse drug reactions, functional decline and cognitive impairment [20]. For these reasons, the management of diabetes in this population is particularly complex and challenging for clinicians and requires a comprehensive geriatric assessment in view of tailoring a personalized approach driven by the principle of “start low and go slow” [21].

In the present cohort, the prevalence of patients with diabetes receiving AD at hospital admission (85%) was quite similar to those reported by a Spanish population-based study. In the latter, both in the overall population (mean age: 70.3 years) and in the sub-cohort of those aged 75 years or older, less than 20% of patients were only recommended an appropriate diet and a healthy lifestyle with no pharmacological treatment [22]. On the contrary, the AD treatment pattern was almost different than those reported by others [23,24,25]. In our study, of all the population included, nearly 62% (=1054/1703) of cases were on monotherapy, while in Spain, less than half of the older population received only one antidiabetic drug [20]. However, our results are in line with those reported by the DISCOVER study, an international program across 38 countries (including Italy) involving patients aged 65 years and older with diabetes who initiate a second line treatment [23], but our results deal with an unselected population.

In our cohort, 36.6% of treated patients received insulin alone or in combination, with only 31.6% of them receiving metformin, whereas in Spain, 28.5% and 72.5% of the oldest patients were prescribed insulin and metformin, respectively [22]. Moreover, a cross-sectional retrospective study in Poland among older patients admitted to geriatric wards reported an overall lower percentage of patients prescribed insulin (32.9%) but higher percentages (56.9%) were found among patients with uncontrolled diabetes [26]. Even the DISCOVER study reported a lower use of insulin (10.1% among European countries) prescribed only as a second-line treatment [23,27]. Herein, the choice to add or to switch the drugs was mainly driven by lack of efficacy of the first line therapy.

In the present cohort, at hospital discharge, the proportion of patients prescribed antidiabetic drugs decreased with an increasing trend over time, and only a few of them (nearly 10%—results not shown) had resumed their previous treatment at the 3-month follow-up. On the other hand, the proportion of patients prescribed with insulin further increased and many of them also remained on insulin within the following three months (53.1%—results not shown). Treatment intensification with insulin was also observed by others, both in the hospital and in other health-care settings. This could be due to an acute clinical condition that led to hospitalization or to a lack or loss of efficacy of the first line therapy [26,27,28].

In the general older population, lifestyle changes and appropriate diet before initiating pharmacological therapy are recommended [4]. When the glycemic target cannot be reached, in order to reduce the risk of diabetes-related complications, a first line therapy with oral antidiabetics with a low risk of hypoglycemia, such as metformin, is advisable. The most important contraindication for the use of metformin is a glomerular filtration rate (GFR) below 30 mL/min [29], which comprises less than 20% of patients in our cohort (data not shown).

During a hospital stay, the clinical guidelines recommend the discontinuation of oral antidiabetic drug therapy. Insulin (basal–bolus or sliding scale) is the preferred treatment because its dosage is easily adjustable [9,30,31,32]. In certain conditions, for example, if patients are able to eat and are in a stable medical condition, the previous home regimen may well be cautiously continued. Anyway, patients should return to their previous effective regimen as soon as possible after the acute episode is resolved [30]. The transition to outpatient care is an important aspect of the management program of patients with diabetes and should be planned at least 1–2 days before the discharge [9]. Notwithstanding recent studies that have provided evidence of the overall safety of starting treatment with insulin in the general T2DM population without increasing the risk of overall mortality and major adverse cardiovascular events [33], the increasing insulin dosage in patients with concomitant heart failure was associated with poor clinical outcomes [34]. In our study, more than 20% of patients with diabetes shifted to insulin during hospital stay from other antidiabetic drugs, and more than 10% started insulin de novo. Among patients starting insulin during hospital stay, nearly 15% were hospitalized for heart failure. Moreover, more than 35% of patients prescribed insulin at hospital discharge were affected by CVD (heart failure and/or ischemic heart disease), thus advocating that a careful assessment among older, frail patients should be carried out before antidiabetic treatment intensification with insulin. Our results suggested that hospital physicians privileged insulin during hospital stay, regardless of the cause of hospital admission, and it seems that they did not review the antidiabetic therapies for these patients.

The use of sulfonylureas should be avoided, particularly in older patients, due to the high risk of hypoglycemia and of drug–drug interactions [35]. The present cohort showed a very low rate of use of this antidiabetic drugs class at hospital admission (9.8% among patients prescribed), with a reduction at hospital discharge and with a clear decreasing trend over time. In the Spanish cohort, prescription with sulfonylureas ranged from 14.3% to 19.9% according to different baseline conditions; in Poland, at hospital discharge, it was 57.5%; and in the overall DISCOVER population, it was 23.5% as first line treatment, increasing to 30.3% as second-line treatment, albeit with marked differences among different geographic areas [22,23,24,25,26]. Only a retrospective cohort of patients hospitalized for diabetes in Australia showed reduced use of this drug (3.8% at hospital discharge) [36]. Our favorable results may be explained in light of the fact that clinicians regularly participating in the REPOSI registry were often sensitized to an optimal management of old patients that leads to an improvement in their knowledge of geriatric pharmacology and risk of drug adverse reactions [37].

Finally, in the 2019 cohort, other classes of antidiabetic drugs, in particular those AD, such as GLP-1-RA and SGLT-2i, that are more recently recommended by clinical guidelines for the management of patients with comorbidities or at high risk of developing cardiovascular complications were prescribed to a handful of patients (i.e., less than 2% of the population).

Patients with T2DM, in addition to their antidiabetic treatments, should receive adjunct cardiovascular therapy (mainly with antihypertensive and lipid-lowering drugs) to reduce their cardiovascular risk and the risk of clinical adverse outcomes. Despite these recommendations, the co-prescription with ACE-I or ARB and/or statins seems to be sub-optimal in our population. Prevalence was slightly lower, but not dissimilar to that found in other Italian cohorts [14,15], but much lower than that found in other countries [36]. In many of our patients who were not co-prescribed ACE-I or ARB at hospital discharge (425/532, 80%), the use of diuretics, calcium channel antagonists and beta-blockers was preferred (data not shown).

About 45% of patients received lipid-lowering drugs; however, no evidence exists on the benefits of starting lipid-lowering drugs in patients aged 80 years or older without manifesting cardio-/cerebro-vascular disease and in those with limited life expectancy and compared to other studies, this percentage seems quite low [13,36,37]. Prescriptions with antiplatelet agents were almost in agreement with the clinical guidelines that recommend aspirin as the first choice for secondary prevention in patients with previous cardio- or cerebrovascular events, but for primary prevention exclusively in those with high cardiovascular risk (e.g., presence of multiple risk factors) [38].

### Strengths and Limitations

The main strength of this study is that the REPOSI is a large sample of nationwide, unselected older patients acutely admitted to internal medicine and geriatric wards in the public hospital setting and is characterized by an extensive description of the health status and medications’ prescriptions, both in the frame of primary care (i.e., at hospital admission) and in the hospital setting.

On the other hand, some social and clinical information was not collected in the framework of the registry. Indeed, in Italy, hospitalization and all services provided during hospital stay are free of charge, so it is not likely that lack of social condition (e.g., billing and health insurance) may affect the results of the present work. However, the lack of specific clinical information (such as the onset of diabetes, and other laboratory tests used to assess disease control (e.g., glycated hemoglobin—HbA1c)), as well as the nutritional status of the patients with diabetes, might be a strong limitation for a better assessment of the medications’ pattern of these patients and may explain the high rate of patients on monotherapy.

## 5. Conclusions

Our results show that the management of older patients with diabetes is complex and often sub-optimal in the hospital setting. Even if those physicians regularly participating in the REPOSI are sensitized to the better pharmacological management of older and frail patients, a great deal of effort and investment should still be expended to better empower the dissemination and implementation of clinical guidelines in different healthcare settings.

## Figures and Tables

**Table 1:** Demographic and clinical characteristics at hospital admission of 1703 patients with diabetes included in the REPOSI registry from 2010 to 2019 and discharged alive.

Characteristics	Diabetic Patients *N* = 1703	N Missing
**Age, years, mean (SD)**	78.3 (7.0)	
**Sex**		
Female	793 (46.6)	
Male	910 (53.4)	
**Year of enrollment**		
2010	324 (19.0)	
2012	336 (19.7)	
2014	269 (15.8)	
2016	175 (10.3)	
2017	224 (13.1)	
2018	161 (9.5)	
2019	214 (12.6)	
**BMI**		215
Underweight (<18.5)	23 (1.5)	
Normal weight (18.5–24.9)	483 (32.5)	
Overweight (25–29.9)	588 (39.5)	
Obesity (≥30)	394 (26.5)	
**Smoke**		59
Never	837 (50.9)	
Ex-smoker	662 (40.3)	
Smoker	145 (8.8)	
**Alcohol**		75
Never	944 (58.0)	
Ex-drinker	191 (11.7)	
Drinker	493 (36.3)	
Social drinker	274 (16.8)	
**Barthel Index**		351
No or negligible dependence (91–100)	635 (47.0)	
Mild dependence (75–90)	302 (22.3)	
Moderate dependence (50–74)	206 (15.2)	
Severe dependence (25–49)	105 (7.8)	
Total dependence (0–24)	104 (7.7)	
**Glucose, mg/dL, mean (SD)**	161.7 (80.7)	47
Low (<100 mg/dL)	293 (17.7)	
Medium (100–125 mg/dL)	316 (19.1)	
High (≥126 mg/dL)	1047 (63.2)	
**Creatinine, mg/dL, mean (SD)**	1.36 (0.9)	19
**GFR, mL/min/1.73 m^2^, mean (SD) ^1^**	55.7 (24.4)	19
**Total cholesterol, mg/dL, mean (SD)**	150.5 (43.0)	462
**Number of drugs at admission ^2^, median (IQR)**	6 (4–8)	
0–1	91 (5.4)	
2–4	491 (28.8)	
5+	1121 (65.8)	
**CIRS—Comorbidity index, mean (SD)**	3.7 (1.9)	
**CIRS—Severity index, mean (SD)**	1.8 (0.3)	
**Co-morbidities ^3^**		
Hypertension	1274 (74.8)	
Ischemic heart disease	582 (34.2)	
CKD	513 (30.1)	
Atrial fibrillation	489 (28.7)	
Heart Failure	353 (20.7)	
Stroke/TIA	340 (20.0)	
Peripheral arterial disease	287 (16.8)	
Dyslipidemia	215 (12.6)	
Dementia	144 (8.5)	

BMI: Body Mass Index; GFR: Glomerular Filtration Rate; CIRS: Cumulative Index Rating Scale; CKD: Chronic Kidney Disease; TIA: Transient Ischemic Attack; SD: Standard Deviation; IQR: Inter-Quartile Range. ^1^ GFR was calculated using the CKD-EPI formula. ^2^ Excluded antidiabetic drugs. ^3^ ICD-9-CM codes for assessing main diagnosis: Hypertension—401 (CIRS item 2); Ischemic heart disease—410–414; CKD—585; Atrial fibrillation—427; Heart failure—428, 402.11; Stroke or TIA—430–438; Peripheral arterial disease—440–441; Dyslipidemia—272; Dementia—290, 294, 310, 331.

**Table 2:** Prevalence of 1703 patients with diabetes treated or not with antidiabetic drugs at hospital admission and discharge.

		Admission *N* = 1703	Discharge *N* = 1703
Untreated		270 (15.8)	394 (23.1)
Treated with monotherapy			
	Insulin monotherapy	482 (28.3)	589 (34.6)
	Metformin monotherapy	328 (19.2)	257 (15.1)
	Sulfonylureas monotherapy	112 (6.6)	69 (4.1)
	Repaglinides monotherapy	107 (6.3)	101 (5.9)
	Other antidiabetic drugs excl. insulin monotherapy ^1^	25 (1.5)	23 (1.4)
Treated with combinations			
	Fixed combination of antidiabetic drugs ^2^	80 (4.7)	38 (2.2)
	Insulins + Metformin	64 (3.8)	63 (3.7)
	Metformin + Sulfonylureas	64 (3.8)	42 (2.5)
	Metformin + Repaglinide	39 (2.3)	26 (1.5)
	Insulins + Other antidiabetic drugs	77 (4.5)	71 (4.2)
	Other combinations of antidiabetic drugs excl. insulin	55 (3.2)	30 (1.7)

^1^ Includes Alpha glucosidase inhibitors, Dipeptidyl Peptidase (DPP-4), Sodium-glucose co-transporter 2 (SGLT2) inhibitors, Thiazolidinediones, Glucagon-like peptide-1 (GLP-1) analogues. ^2^ Includes metformin and sulfonylureas; metformin and sitagliptin; metformin and vildagliptin; phenformin and sulfonylureas; metformin and pioglitazone; glimepiride and pioglitazone.

## Data Availability

Research data can be shared upon request to the study team.

## Supplementary Materials

The following supporting information can be downloaded at: https://www.mdpi.com/article/10.3390/ijerph20032607/s1, Table S1: Cause of hospital admission for 1703 patients with diabetes included in the REPOSI registry; Table S2: Prevalence of patients treated or not with different antidiabetic drugs as monotherapy or combination at admission by years; Table S3: Prevalence of patients treated or not with different antidiabetic drugs as monotherapy or combination at discharge by years; Table S4: Prevalence of treated patients with diabetes using or not cardiovascular drugs at admission by years; Table S5: Prevalence of treated patients with diabetes using or not cardiovascular drugs at discharge by years.

## Appendix A

Investigators and co-authors of the REPOSI (REgistro POliterapie SIMI, Società Italiana di Medicina Interna) Study Group are as follows:

**Steering Committee:** Pier Mannuccio Mannucci (*Chair*) (*Fondazione IRCCS Cà Granda Ospedale Maggiore Policlinico, Milano*), Alessandro Nobili (*co-chair*) (*Istituto di Ricerche Farmacologiche Mario Negri IRCCS, Milano*), Giorgio Sesti (*Presidente SIMI*), Antonello Pietrangelo (*Direttore CRIS—SIMI*), Francesco Perticone (*Università Magna Grecia Policlinico Mater Domini, Catanzaro*) Francesco Violi (*Policlinico Umberto I, Roma*), Gino Roberto Corazza, (*IRCCS Policlinico San Matteo di Pavia, Pavia*), Salvatore Corrao (*ARNAS Civico, Di Cristina, Benfratelli, DiBiMIS, Università di Palermo, Palermo*), Alessandra Marengoni (*Spedali Civili di Brescia, Brescia*), Francesco Salerno (*IRCCS Policlinico San Donato Milanese, Milano*), Matteo Cesari (*Fondazione Maugeri, Milano*), Mauro Tettamanti, Luca Pasina, Carlotta Franchi (*Istituto di Ricerche Farmacologiche Mario Negri IRCCS, Milano*).
**Clinical Data Monitoring and Revision:** Carlotta Franchi, Alessio Novella, Mauro Tettamanti, Gabriella Miglio (*Istituto di Ricerche Farmacologiche Mario Negri IRCCS, Milano*).
**Database Management and Statistics:** Mauro Tettamanti, Alessia Antonella Galbussera, Ilaria Ardoino, Alessio Novella (*Istituto di Ricerche Farmacologiche Mario Negri IRCCS, Milano*).

**Investigators:**

- Domenico Prisco, Elena Silvestri, Giacomo Emmi, Alessandra Bettiol, Irene Mattioli (*Azienda Ospedaliero Universitaria Careggi Firenze, SOD Medicina Interna Interdisciplinare*);
- Gianni Biolo, Michela Zanetti, Giacomo Bartelloni, Michele Zaccari, Massimiliano Chiuch (*Azienda Sanitaria Universitaria Integrata di Trieste, Clinica Medica Generale e Terapia Medica*);
- Massimo Vanoli, Giulia Grignani, Edoardo Alessandro Pulixi (*Azienda Ospedaliera della Provincia di Lecco, Ospedale di Merate, Lecco, Medicina Interna*);
- Matteo Pirro, Graziana Lupattelli, Vanessa Bianconi, Riccardo Alcidi, Alessia Giotta, Massimo R. Mannarino (*Azienda Ospedaliera Santa Maria della Misericordia, Perugia, Medicina Interna, Angiologia Malattie da Arteriosclerosi*);
- Domenico Girelli, Fabiana Busti, Giacomo Marchi (*Azienda Ospedaliera Universitaria Integrata di Verona, Verona, Medicina Generale e Malattie Aterotrombotiche e Degenerative*);
- Mario Barbagallo, Ligia Dominguez, Vincenza Beneduce, Federica Cacioppo (*Azienda Ospedaliera Universitaria Policlinico Giaccone Policlinico di Palermo, Palermo, Unità Operativa di Geriatria e Lungodegenza*);
- Salvatore Corrao, Giuseppe Natoli, Salvatore Mularo, Massimo Raspanti, Christiano Argano, Federica Cavallaro (*A.R.N.A.S. Civico, Di Cristina, Benfratelli, Palermo, UOC Medicina Interna ad Indirizzo Geriatrico-Riabilitativo*);
- Marco Zoli, Maria Laura Matacena, Giuseppe Orio, Eleonora Magnolfi, Giovanni Serafini, Angelo Simili, Mattia Brunori, Ilaria Lazzari, Angelo Simili (*Azienda Ospedaliera Universitaria Policlinico S. Orsola-Malpighi, Bologna, Unità Operativa di Medicina Interna Zoli*);
- Maria Domenica Cappellini, Giovanna Fabio, Margherita Migone De Amicis, Giacomo De Luca, Natalia Scaramellini, Valeria Di Stefano, Simona Leoni, Sonia Seghezzi, Alessandra Danuto Di Mauro, Diletta Maira, Marta Mancarella (*Fondazione IRCCS Cà Granda Ospedale Maggiore Policlinico, Milano, Unità Operativa Medicina Interna IA*);
- Tiziano Lucchi, Paolo Dionigi Rossi, Marta Clerici, Simona Leoni, Alessandra Danuta Di Mauro, Giulia Bonini, Federica Conti, Silvia Prolo, Maddalena Fabrizi, Miriana Martelengo, Giulia Vigani (*Fondazione IRCCS Cà Granda Ospedale Maggiore Policlinico, Milano, Geriatria*);
- Antonio Di Sabatino, Emanuela Miceli, Marco Vincenzo Lenti, Martina Pisati, Costanza Caccia Dominioni, Lavinia Pitotti, Donatella Padula (*IRCCS Policlinico San Matteo di Pavia, Pavia, Clinica Medica I, Reparto 11*);
- Roberto Pontremoli, Valentina Beccati, Giulia Nobili, Giovanna Leoncini, Jacopo Alberto, Federico Cattaneo (*IRCCS Azienda Ospedaliera Universitaria San Martino-IST di Genova, Genova, Clinica di Medicina Interna 2*);
- Luigi Anastasio, Lucia Sofia, Maria Carbone (*Ospedale Civile Jazzolino di Vibo Valentia, Vibo Valentia, Medicina Generale*);
- Francesco Cipollone, Maria Teresa Guagnano, Ilaria Rossi, Emanuele Valeriani, Damiani D’Ardes, Lucia Esposito, Simona Sestili, Ermanno Angelucci (*Ospedale Clinicizzato SS. Annunziata, Chieti, Clinica Medica*);
- Gerardo Mancuso, Daniela Calipari, Mosè Bartone (*Ospedale Giovanni Paolo II Lamezia Terme, Catanzaro, Unità Operativa Complessa Medicina Interna*);
- Giuseppe Delitala, Maria Berria, Alessandro Delitala (*Azienda ospedaliera-universitaria di Sassari, Clinica Medica*);
- Maurizio Muscaritoli, Alessio Molfino, Enrico Petrillo, Antonella Giorgi, Christian Gracin, Giovanni Imbimbo (*Policlinico Umberto I, Sapienza Università di Roma, Medicina Interna e Nutrizione Clinica Policlinico Umberto I*);
- Giuseppe Zuccalà, Gabriella D’Aurizio (*Policlinico Universitario A. Gemelli, Roma, Roma, Unità Operativa Complessa Medicina d’Urgenza e Pronto Soccorso*)
- Giuseppe Romanelli, Alessandra Marengoni, Andrea Volpini, Daniela Lucente, Francesca Manzoni, Annalisa Pirozzi, Alberto Zucchelli (*Unità Operativa Complessa di Medicina I a indirizzo geriatrico, Spedali Civili, Montichiari, Brescia*);
- Antonio Picardi, Umberto Vespasiani Gentilucci, Paolo Gallo, Chiara Dell’Unto (*Università Campus Bio-Medico, Roma, Medicina Clinica-Epatologia*);
- Giuseppe Bellelli, Maurizio Corsi, Cesare Antonucci, Chiara Sidoli, Giulia Principato, Alessandra Bonfanti, Hajnalka Szabo, Paolo Mazzola, Andrea Piazzoli, Maurizio Corsi (*Università degli studi di Milano-Bicocca Ospedale S. Gerardo, Monza, Unità Operativa di Geriatria*);
- Franco Arturi, Elena Succurro, Bruno Tassone, Federica Giofrè (*Università degli Studi Magna Grecia, Policlinico Mater Domini, Catanzaro, Unità Operativa Complessa di Medicina Interna*);
- Maria Grazia Serra, Maria Antonietta Bleve (*Azienda Ospedaliera “Cardinale Panico” Tricase, Lecce, Unità Operativa Complessa Medicina*);
- Antonio Brucato, Teresa De Falco, Enrica Negro, Martino Brenna, Lucia Trotta (*ASST Fatebenefratelli—Sacco, Milano, Medicina Interna*);
- Maria Luisa Randi, Fabrizio Fabris, Irene Bertozzi, Giulia Bogoni, Maria Victoria Rabuini, Tancredi Prandini, Francesco Ratti, Chiara Zurlo, Lorenzo Cerruti, Elisabetta Cosi (*Azienda Ospedaliera Università di Padova, Padova, Clinica Medica I*);
- Roberto Manfredini, Fabio Fabbian, Benedetta Boari, Alfredo De Giorgi, Ruana Tiseo (*Azienda Ospedaliera—Universitaria Sant’Anna, Ferrara, Unità Operativa Clinica Medica*);
- Giuseppe Paolisso, Maria Rosaria Rizzo, Claudia Catalano, Irene Di Meo (*Azienda Ospedaliera Universitaria della Seconda Università degli Studi di Napoli, Napoli, VI Divisione di Medicina Interna e Malattie Nutrizionali dell’Invecchiamento*);
- Claudio Borghi, Enrico Strocchi, Eugenia Ianniello, Mario Soldati, Silvia Schiavone, Alessio Bragagni, Francesca Giulia Leoni, Valeria De Sando, Sara Scarduelli, Michela Cammarosano, Ilenia Pareo (*Azienda Ospedaliera Universitaria Policlinico S. Orsola-Malpighi, Bologna, Unità Operativa di Medicina Interna Borghi*);
- Carlo Sabbà, Francesco Saverio Vella, Patrizia Suppressa, Giovanni Michele De Vincenzo, Alessio Comitangelo, Emanuele Amoruso, Carlo Custodero, Giuseppe Re, Andrea Schilardi, Francesca Loparco (*Azienda Ospedaliero-Universitaria Consorziale Policlinico di Bari, Bari, Medicina Interna Universitaria C. Frugoni*);
- Luigi Fenoglio, Andrea Falcetta, Alessia Valentina Giraudo, Salvatore D’Aniano (*Azienda Sanitaria Ospedaliera Santa Croce e Carle di Cuneo, Cuneo, S. C. Medicina Interna*);
- Anna L. Fracanzani, Silvia Tiraboschi, Annalisa Cespiati, Giovanna Oberti, Giordano Sigon, Felice Cinque (*Fondazione IRCCS Cà Granda Ospedale Maggiore Policlinico, Milano, UOC Medicina Generale ad Indirizzo Metabolico*);
- Flora Peyvandi, Raffaella Rossio, Giulia Colombo, Pasquale Agosti, Erica Pagliaro (*Fondazione IRCCS Cà Granda Ospedale Maggiore Policlinico, Milano, Medicina Interna 2, Ematologia non tumorale e Coagulopatie*);
- Canetta Ciro, Valter Monzani, Valeria Savojardo, Giuliana Ceriani, Christian Folli (*Fondazione IRCCS Cà Granda Ospedale Maggiore Policlinico, Milano, Medicina Interna Alta Intensità di Cure*);
- Francesco Salerno, Giada Pallini (*IRCCS Policlinico San Donato e Università di Milano, San Donato Milanese, Medicina Interna*);
- Fabrizio Montecucco, Luciano Ottonello, Lara Caserza, Giulia Vischi, Salam Kassem, Luca Liberale (*IRCCS Ospedale Policlinico San Martino e Università di Genova, Genova, Clinica Medica 1, Medicina Interna e Specialità Mediche*);
- Nicola Lucio Liberato, Tiziana Tognin (*ASST di Pavia, UOSD Medicina Interna, Ospedale di Casorate Primo, Pavia*);
- Francesco Purrello, Antonino Di Pino, Salvatore Piro (*Ospedale Garibaldi Nesima, Catania, Unità Operativa Complessa di Medicina Interna*);
- Renzo Rozzini, Lina Falanga, Maria Stella Pisciotta, Francesco Baffa Bellucci, Stefano Buffelli, Camillo Ferrandina, Francesca Mazzeo, Elena Spazzini, Giulia Cono, Giulia Cesaroni (*Ospedale Poliambulanza, Brescia, Medicina Interna e Geriatria*);
- Giuseppe Montrucchio, Paolo Peasso, Edoardo Favale, Cesare Poletto, Carl Margaria, Maura Sanino (*Dipartimento di Scienze Mediche, Università di Torino, Città della Scienza e della Salute, Torino, Medicina Interna 2 Unità Indirizzo d’Urgenza*);
- Francesco Violi, Ludovica Perri (*Policlinico Umberto I, Roma, Prima Clinica Medica*);
- Luigina Guasti, Luana Castiglioni, Andrea Maresca, Alessandro Squizzato, Leonardo Campiotti, Alessandra Grossi, Roberto Davide Diprizio, Francesco Dentali (*Università degli Studi dell’Insubria, Ospedale di Circolo e Fondazione Macchi, Varese, Medicina e Geriatria*);
- Marco Bertolotti, Chiara Mussi, Giulia Lancellotti, Maria Vittoria Libbra, Matteo Galassi, Yasmine Grassi, Alessio Greco, Elena Bigi, Elisa Pellegrini, Laura Orlandi, Giulia Dondi, Lucia Carulli (*Università di Modena e Reggio Emilia, Azienda Ospedaliero-Universitaria di Modena; Ospedale Civile di Baggiovara, Unità Operativa di Geriatria*);
- Angela Sciacqua, Maria Perticone, Rosa Battaglia, Raffaele Maio, Aleandra Scozzafava, Valentino Condoleo, Tania Falbo, Lidia Colangelo; Marco Filice, Elvira Clausi (*Università Magna Grecia Policlinico Mater Domini, Catanzaro, Unità Operativa Malattie Cardiovascolari Geriatriche*);
- Vincenzo Stanghellini, Eugenio Ruggeri, Sara del Vecchio, Ilaria Benzoni (*Dipartimento di Scienze Mediche e Chirurgiche, Unità Operativa di Medicina Interna, Università degli Studi di Bologna/Azienda Ospedaliero-Universitaria S.Orsola-Malpighi, Bologna*);
- Andrea Salvi, Roberto Leonardi, Giampaolo Damiani (*Spedali Civili di Brescia, U.O. 3a Medicina Generale*);
- Gianluca Moroncini, William Capeci, Massimo Mattioli, Giuseppe Pio Martino, Lorenzo Biondi, Pietro Pettinari, Monica Ormas, Emanuele Filippini, Devis Benfaremo, Roberto Romiti (*Clinica Medica, Azienda Ospedaliera Universitaria-Ospedali Riuniti di Ancona*);
- Riccardo Ghio, Anna Dal Col (*Azienda Ospedaliera Università San Martino, Genova, Medicina III*);
- Salvatore Minisola, Luciano Colangelo, Mirella Cilli, Giancarlo Labbadia (*Policlinico Umberto I, Roma, SMSC03—Medicina Interna F e Malattie Metaboliche dell’osso*);
- Antonella Afeltra, Benedetta Marigliano, Maria Elena Pipita (*Policlinico Campus Biomedico Roma, Roma, Medicina Clinica*);
- Pietro Castellino, Luca Zanoli, Alfio Gennaro, Agostino Gaudio, Samuele Pignataro (*Azienda Ospedaliera Universitaria Policlinico—V. Emanuele, Catania, Dipartimento di Medicina*);
- Francesca Mete, Miriam Gino (*Ospedale degli Infermi di Rivoli, Torino, Medicina Interna*);
- Guido Moreo, Silvia Prolo, Gloria Pina (*Clinica San Carlo Casa di Cura Polispecialistica, Paderno Dugnano, Milano, Unità Operativa di Medicina Generale Emilio Bernardelli*);
- Alberto Ballestrero, Fabio Ferrando, Roberta Gonella, Domenico Cerminara, Paolo Setti, Chiara Traversa, Camilla Scarsi (*Clinica Di Medicina Interna ad Indirizzo Oncologico, Azienda Ospedaliera Università San Martino di Genova*);
- Bruno Graziella, Stefano Baldassarre, Salvatore Fragapani, Gabriella Gruden (*Medicina Interna III, Ospedale S. Giovanni Battista Molinette, Torino*);
- Franco Berti, Giuseppe Famularo, Patrizia Tarsitani (*Azienda Ospedaliera San Camillo Forlanini, Roma, Medicina Interna II*);
- Roberto Castello, Michela Pasino (*Ospedale Civile Maggiore Borgo Trento, Verona, Medicina Generale e Sezione di Decisione Clinica*);
- Marcello Giuseppe Maggio Gian Paolo Ceda, Simonetta Morganti, Andrea Artoni, Margherita Grossi (*Azienda Ospedaliero Universitaria di Parma, U.O.C Clinica Geriatrica*);
- Stefano Del Giacco, Davide Firinu, Giulia Costanzo, Giacomo Argiolas, Giovanni Paoletti, Francesca Losa (*Policlinico Universitario Duilio Casula, Azienda Ospedaliero-Universitaria di Cagliari, Cagliari, Medicina Interna, Allergologia ed Immunologia Clinica*);
- Giuseppe Montalto, Anna Licata, Filippo Alessandro Montalto (*Azienda Ospedaliera Universitaria Policlinico Paolo Giaccone, Palermo, UOC di Medicina Interna*);
- Francesco Corica, Giorgio Basile, Antonino Catalano, Federica Bellone, Concetto Principato (*Azienda Ospedaliera Universitaria Policlinico G. Martino, Messina, Unità Operativa di Geriatria*);
- Lorenzo Malatino, Benedetta Stancanelli, Valentina Terranova, Salvatore Di Marca, Rosario Di Quattro, Lara La Malfa, Rossella Caruso (*Azienda Ospedaliera per l’Emergenza Cannizzaro, Catania, Clinica Medica Università di Catania*);
- Patrizia Mecocci, Carmelinda Ruggiero, Virginia Boccardi (*Università degli Studi di Perugia-Azienda Ospedaliera S.M. della Misericordia, Perugia, Struttura Complessa di Geriatria*);
- Tiziana Meschi, Andrea Ticinesi, Antonio Nouvenne (*Azienda Ospedaliera Universitaria di Parma, U.O Medicina Interna e Lungodegenza Critica*);
- Pietro Minuz, Luigi Fondrieschi, Giandomenico Nigro Imperiale, Sarah Morellini (*Azienda Ospedaliera Universitaria Verona, Policlinico GB Rossi, Verona, Medicina Generale per lo Studio ed il Trattamento dell’Ipertensione Arteriosa*);
- Mario Pirisi, Gian Paolo Fra, Daniele Sola, Mattia Bellan (*Azienda Ospedaliera Universitaria Maggiore della Carità, Medicina Interna 1*);
- Roberto Quadri, Erica Larovere, Marco Novelli (*Ospedale di Ciriè, ASL TO4, Torino, S.C. Medicina Interna*);
- Emilio Simeone, Rosa Scurti, Fabio Tolloso (*Ospedale Spirito Santo di Pescara, Geriatria*);
- Roberto Tarquini, Alice Valoriani, Silvia Dolenti, Giulia Vannini (*Ospedale San Giuseppe, Empoli, USL Toscana Centro, Firenze, Medicina Interna I*);
- Riccardo Volpi, Pietro Bocchi, Alessandro Vignali (*Azienda Ospedaliera Universitaria di Parma, Clinica e Terapia Medica*);
- Sergio Harari, Chiara Lonati, Federico Napoli, Italia Aiello (*Ospedale San Giuseppe Multimedica Spa, U.O. Medicina Generale*);
- Francesco Purrello, Antonino Di Pino (*Ospedale Garibaldi-Nesima–Catania, U.O.C Medicina Interna*);
- Teresa Salvatore, Lucio Monaco, Carmen Ricozzi (*Policlinico Università della Campania L. Vanvitelli, UOC Medicina Interna*);
- Alberto Pilotto, Ilaria Indiano, Federica Gandolfo (*Ente Ospedaliero Ospedali Galliera Genova, SC Geriatria Dipartimento Cure Geriatriche, Ortogeriatria e Riabilitazione*)
- Franco Laghi Pasini, Pier Leopoldo Capecchi (*Azienda Ospedaliera Universitaria Senese, Siena, Unità Operativa Complessa Medicina 2*);
- Ranuccio Nuti, Roberto Valenti, Martina Ruvio, Silvia Cappelli, Alberto Palazzuoli (*Azienda Ospedaliera Università Senese, Siena, Medicina Interna I*);
- Mauro Bernardi, Silvia Li Bassi, Luca Santi, Giacomo Zaccherini (*Azienda Ospedaliera Policlinico Sant’Orsola-Malpighi, Bologna, Semeiotica Medica Bernardi*);
- Vittorio Durante, Daniela Tirotta, Giovanna Eusebi (*Ospedale di Cattolica, Rimini, Medicina Interna*);
- Marco Cattaneo, Maria Valentina Amoruso, Paola Fracasso, Cristina Fasolino (*Azienda ospedaliera San Paolo, Milano, Medicina III*);
- Moreno Tresoldi, Enrica Bozzolo, Sarah Damanti (*IRCCS Ospedale San Raffaele–Milano, Medicina Generale e delle Cure Avanzate*);
- Massimo Porta, Miriam Gino (*AOU Città della Salute e della Scienza di Torino–Torino, Medicina Interna 1U*).
