# Supplementary material for: Antidiabetic Drug Prescription Pattern in Hospitalized Older Patients with Diabetes
Source: Int J Environ Res Public Health. 2023 Jan 31;20(3):2607. doi: 10.3390/ijerph20032607 (PMC9915986; doi:10.3390/ijerph20032607)
Supplement: Supplementary file 1 [file ijerph-20-02607-s001.zip › ijerph-2114144-supplementary.pdf]

Table S1. Cause of hospital admission for 1703 patients with diabetes included in the REPOSI registry

Cause of hospital admission	N (%)
**Heart diseases**	
Heart failure	**178 (10.5)**
Atrial fibrillation	**32 (1.9)**
**Symptoms, signs, and ill-defined conditions**	
Dyspnea	**171 (10.0)**
Syncope	**140 (8.2)**
Diarrhea, nausea and vomiting	**23 (1.4)**
Abdominal pain	**51 (3.0)**
**Diabetes**	
Diabetes	**73 (4.3)**
Hypoglycemia	**17 (1.0)**
**Pneumonia**	
Bacterial pneumonia	**60 (3.5)**
Pneumonia (organism unspecified)	**24 (1.4)**
Bronchopneumonia	**20 (1.2)**
**Other Respiratory diseases**	**54 (3.2)**
**COPD**	**36 (2.1)**
**Blood diseases**	
Anemia	**70 (4.1)**
Iron deficiency anemia	**36 (2.1)**
**Cerebrovascular diseases**	
TIA	**23 (1.4)**
Cerebral thrombosis	**22 (1.3)**
**Other**	**673 (39.5)**

COPD: Chronic Obstructive Pulmonary Disease; TIA: Transient Ischemic Attack.
ICD-9-CM code for assessing cause of hospital admission: Heart Failure: 428; Atrial fibrillation: 427; Dyspnea: 786; Syncope: 780; Diarrhea, nausea and vomiting: 787; Abdominal pain: 789; Diabetes: 250; Hypoglycemia: 251; Bacterial pneumonia: 482; Pneumonia (organism unspecified): 486; Bronchopneumonia:485; Other respiratory diseases: 518; COPD: 491; Anemia: 285; Iron deficiency anemia: 280; TIA: 435; Cerebral Thrombosis: 434; Other included specific conditions with a prevalence less than 2%

Table S2. Prevalence of patients treated or not with different antidiabetic drugs as monotherapy or combination at admission by years

	2010<br>N=324	2012<br>N=336	2014<br>N=269	2016<br>N=175	2017<br>N=224	2018<br>N=161	2019<br>N=214
Untreated, n (%)	46 (14.2)	56 (16.7)	32 (11.9)	22 (12.6)	37 (16.5)	36 (22.4)	41 (19.1)
Insulin monotherapy	85 (26.2)	85 (25.3)	85 (31.6)	54 (30.9)	72 (32.1)	40 (24.8)	61 (28.5)
Metformin monotherapy	51 (15.7)	58 (17.3)	59 (33.7)	38 (21.7)	41 (18.3)	33 (20.5)	48 (22.4)
Sulfonylureas monotherapy	34 (10.5)	26 (7.7)	17 (9.7)	11 (6.3)	9 (4.0)	8 (5.0)	7 (3.3)
Repaglinides monotherapy	21 (6.5)	35 (10.4)	12 (4.5)	11 (6.3)	15 (6.7)	7 (4.3)	6 (2.8)
Other antidiabetic drugs excl. Insulins monotherapy[1]	1 (0.3)	3 (0.9)	2 (0.7)	4 (2.3)	4 (1.8)	3 (1.9)	8 (3.7)
Fixed combination of antidiabetic drugs[2]	32 (9.9)	18 (5.3)	11 (4.1)	6 (3.4)	5 (2.2)	5 (3.1)	3 (1.4)
Insulins+Other antidiabetic drugs	17 (5.2)	12 (3.5)	14 (5.2)	10 (5.7)	11 (4.9)	1 (0.6)	12 (5.6)
Insulins+Metformin	10 (3.1)	12 (3.6)	6 (2.2)	7 (4.0)	8 (3.6)	8 (5.0)	13 (6.1)
Metformin+Sulfonylureas	11 (3.4)	15 (4.5)	14 (5.2)	2 (1.1)	7 (3.1)	10 (6.2)	5 (2.3)
Metformin+Repaglinide	8 (2.5)	7 (2.1)	8 (3.0)	6 (3.4)	5 (2.3)	3 (1.9)	2 (0.9)
Other combination of antidiabetic drugs excl. Insulins	8 (2.5)	9 (2.7)	9 (3.3)	4 (2.3)	10 (4.5)	7 (4.3)	8 (3.7)

[1] Includes: Alpha glucosidase inhibitors, Dipeptidyl Peptidase (DPP-4), Sodium-glucose co-transporter 2 (SGLT2) inhibitors, Thiazolidinediones, Glucagon-like peptide-1 (GLP-1) analogues

[2] Includes: metformin and sulfonylureas; metformin and sitagliptin; metformin and vildagliptin; phenformin and sulfonylureas; metformin and pioglitazone; glimepiride and pioglitazone

Table S3. Prevalence of patients treated or not with different antidiabetic drugs as monotherapy or combination at discharge by years

	2010<br>N=324	2012<br>N=336	2014<br>N=269	2016<br>N=175	2017<br>N=224	2018<br>N=161	2019<br>N=214
Untreated, n (%)	46 (14.2)	68 (20.2)	61 (22.7)	52 (29.7)	61 (27.2)	48 (29.8)	58 (27.1)
Insulin monotherapy	107 (33.0)	107 (31.8)	99 (36.8)	63 (36.0)	89 (39.7)	52 (32.3)	72 (33.6)
Metformin monotherapy	48 (14.8)	49 (14.6)	40 (14.9)	30 (17.1)	29 (12.9)	27 (16.8)	34 (15.9)
Sulfonylureas monotherapy	28 (8.6)	16 (4.8)	9 (3.3)	5 (2.9)	4 (1.8)	4 (2.5)	3 (1.4)
Repaglinides monotherapy	23 (7.1)	35 (10.4)	18 (6.7)	5 (2.9)	11 (4.9)	7 (4.3)	2 (0.9)
Other antidiabetic drugs excl. Insulins monotherapy[1]	1 (0.3)	3 (0.9)	3 (1.1)	2 (1.1)	4 (1.8)	3 (1.9)	7 (3.3)
Fixed combination of antidiabetic drugs[2]	17 (5.2)	7 (2.1)	6 (2.2)	2 (1.1)	3 (1.3)	2 (1.2)	1 (0.5)
Insulins+Other antidiabetic drugs	18 (5.6)	13 (3.8)	12 (4.5)	7 (4.0)	5 (2.3)	2 (1.2)	14 (6.5)
Insulins+Metformin	14 (4.3)	12 (3.6)	7 (2.6)	5 (2.9)	7 (3.1)	7 (4.3)	11 (5.1)
Metformin+Sulfonylureas	11 (3.4)	9 (2.7)	10 (3.7)	0	4 (1.8)	5 (3.2)	3 (1.4)
Metformin+Repaglinide	7 (2.2)	10 (3.0)	3 (1.1)	3 (1.7)	2 (0.9)	0	1 (0.5)
Other combination of antidiabetic drugs excl. Insulins	4 (1.3)	7 (2.1)	1 (0.4)	1 (0.6)	5 (2.3)	4 (2.5)	8 (3.8)

[1] Includes: Alpha glucosidase inhibitors, Dipeptidyl Peptidase (DPP-4), Sodium-glucose co-transporter 2 (SGLT2) inhibitors, Thiazolidinediones, Glucagon-like peptide-1 (GLP-1) analogues

[2] Includes: metformin and sulfonylureas; metformin and sitagliptin; metformin and vildagliptin; phenformin and sulfonylureas; metformin and pioglitazone; glimepiride and pioglitazone

Table S4. Prevalence of treated patients with diabetes using or not cardiovascular drugs at admission by years

Cardiovascular drugs	2010 N=278	2012 N=280	2014 N=237	2016 N=153	2017 N=187	2018 N=125	2019 N=173
Not treated, n (%)	62 (22.3)	50 (17.9)	44 (18.6)	25 (16.3)	23 (12.3)	23 (18.4)	33 (19.1)
ACE-I/ARBs (C09), n (%)	45 (16.2)	62 (22.1)	46 (19.4)	22 (14.4)	27 (14.4)	25 (20.0)	35 (20.2)
Lipid-lowering drugs (C10), n (%)	14 (5.0)	18 (6.4)	8 (3.4)	10 (6.5)	13 (6.9)	6 (4.8)	18 (10.4)
Antiplatelet (B01AC), n (%)	18 (6.5)	24 (8.6)	18 (7.6)	12 (7.8)	19 (10.2)	10 (8.0)	10 (5.8)
ACE-I (C09) + Lipid-lowering drugs (C10), n (%)	34 (12.2)	15 (5.4)	25 (10.5)	19 (13.9)	26 (13.9)	20 (16.0)	23 (13.3)
ACE-I/ARBs (C09) + Antiplatelet (B01AC), n (%)	43 (15.5)	44 (15.7)	28 (11.8)	23 (15.0)	21 (11.2)	11 (8.8)	11 (6.4)
Lipid-lowering drugs (C10) + Antiplatelet (B01AC), n (%)	14 (5.0)	13 (4.4)	14 (5.9)	19 (12.4)	15 (8.0)	12 (9.6)	16 (9.2)
All three, n (%)	48 (17.3)	54 (19.3)	54 (22.8)	23 (15.0)	43 (23.0)	18 (14.4)	27 (15.6)

Table S5. Prevalence of treated patients with diabetes using or not cardiovascular drugs at discharge by years

Cardiovascular drugs	2010 N=278	2012 N=280	2014 N=237	2016 N=153	2017 N=187	2018 N=125	2019 N=173
Not treated, n (%)	55 (19.8)	50 (18.7)	39 (18.8)	20 (16.3)	23 (14.1)	24 (21.2)	35 (22.4)
ACE-I/ARBs (C09), n (%)	42 (15.1)	51 (19.0)	31 (14.9)	13 (10.6)	24 (14.7)	23 (20.3)	28 (17.9)
Lipid-lowering drugs (C10), n (%)	14 (5.0)	18 (6.7)	10 (4.8)	11 (8.9)	14 (8.6)	7 (6.2)	14 (9.0)
Antiplatelet (B01AC), n (%)	21 (7.6)	24 (9.0)	19 (9.1)	11 (8.9)	11 (6.7)	4 (3.5)	9 (5.8)
ACE-I (C09) + Lipid-lowering drugs (C10), n (%)	29 (10.4)	20 (7.5)	22 (10.6)	15 (12.2)	23 (14.1)	16 (14.2)	23 (14.7)
ACE-I/ARBs (C09) + Antiplatelet (B01AC), n (%)	47 (16.9)	37 (13.8)	26 (12.5)	19 (15.5)	13 (8.0)	10 (8.9)	6 (3.8)
Lipid-lowering drugs (C10) + Antiplatelet (B01AC), n (%)	19 (6.8)	11 (4.1)	17 (8.2)	12 (9.8)	10 (6.1)	14 (12.4)	16 (10.3)
All three, n (%)	51 (18.4)	57 (21.3)	44 (21.1)	22 (17.9)	45 (27.6)	15 (13.3)	25 (16.0)
